# Efficacy of Zwolle Score in Predicting Outcomes of Patients With ST-Segment Elevation Myocardial Infarction

**DOI:** 10.1016/j.jscai.2024.102389

**Published:** 2024-09-26

**Authors:** Akshay Machanahalli Balakrishna, Khansa Ahmad, Melvin G. Joice, Alexander G. Truesdell, Syed Tanveer Rab, Jinnette Dawn Abbott, Saraschandra Vallabhajosyula

**Affiliations:** a Division of Cardiovascular Medicine, Department of Medicine, Creighton University School of Medicine, Omaha, Nebraska; b Division of Cardiology, Department of Medicine, Warren Alpert Medical School of Brown University, Providence, Rhode Island; c Virginia Heart/Inova Schar Heart and Vascular Institute, Falls Church, Virginia; d Division of Cardiology, Department of Medicine, Emory University School of Medicine, Atlanta, Georgia; e Brown University Health Cardiovascular Institute, Providence, Rhode Island

**Keywords:** early discharge, mortality, percutaneous coronary intervention, ST-segment elevation myocardial infarction, Zwolle risk score

Despite steady improvements in the management and outcomes of patients presenting with ST-segment elevation myocardial infarction (STEMI), there are limited data regarding the ideal length of stay.^1^^,^^2^ Guidelines suggest that patients should be monitored for post-MI complications for at least 24 to 48 hours in the hospital. There is insufficient evidence to suggest that a longer stay in the hospital leads to better patient outcomes.^1^^,^^3^ Prioritizing early discharge of patients at low risk for significant adverse outcomes following primary percutaneous coronary intervention (PCI) for STEMI has advantages including decreasing the risk of nosocomial complications and improving resource utilization including cost and staffing, and optimization of inpatient capacity. Numerous risk stratification score models are available to predict short-term mortality.^1^, ^2^, ^3^, ^4^ The Zwolle risk score (ZRS) was developed to assess the risk of complications, including early mortality, after PCI in patients with STEMI.^1^^,^^2^^,^^5^, ^6^, ^7^ ZRS has been validated in multiple studies to be safe, economical, and helpful in identifying patients who can be discharged early.^1^^,^^2^ In that context, we performed a meta-analysis to determine the predictive accuracy of the ZRS for short-term adverse outcomes in STEMI patients.

A systematic review was conducted in PubMed, EMBASE, and ClinicalTrials.gov from inception through July 2024. The study protocol did not require institutional review board approval because publicly available data were used. All studies that utilized ZRS to risk stratify STEMI patients and reported the C-statistic for predicting the outcomes of interest including short-term mortality (defined as in-hospital or mortality ≤30 days) and 30-day major adverse cardiovascular events (MACE) were included in the meta-analysis. The definition of MACE was slightly variable in different studies and was defined as a composite of cardiac death, stroke, recurrent myocardial infarction, and congestive cardiac failure by Lim et al^3^ and as a composite of mortality, reinfarction, repeat revascularization, cerebrovascular events, any bleeding event, or unplanned hospitalization due to heart failure by Ali Shah et al.^6^ Low risk was defined as ZRS ≤3, and high risk as ZRS ≥4 in the included studies. Two reviewers (A.M.B., K.A.) independently retrieved relevant information. Any differences between the reviewers were resolved by consultation with other authors (M.G.J., S.V.). Due to the anticipated heterogeneity, the use of a random-effects model was used to pool C-statistics and corresponding SDs from each study. The included studies’ quality and risk of bias were evaluated using the Newcastle-Ottawa scale quality score. All analyses were conducted using Open Meta-Analyst-0.1503 software (Brown University).

**Figure 1:**
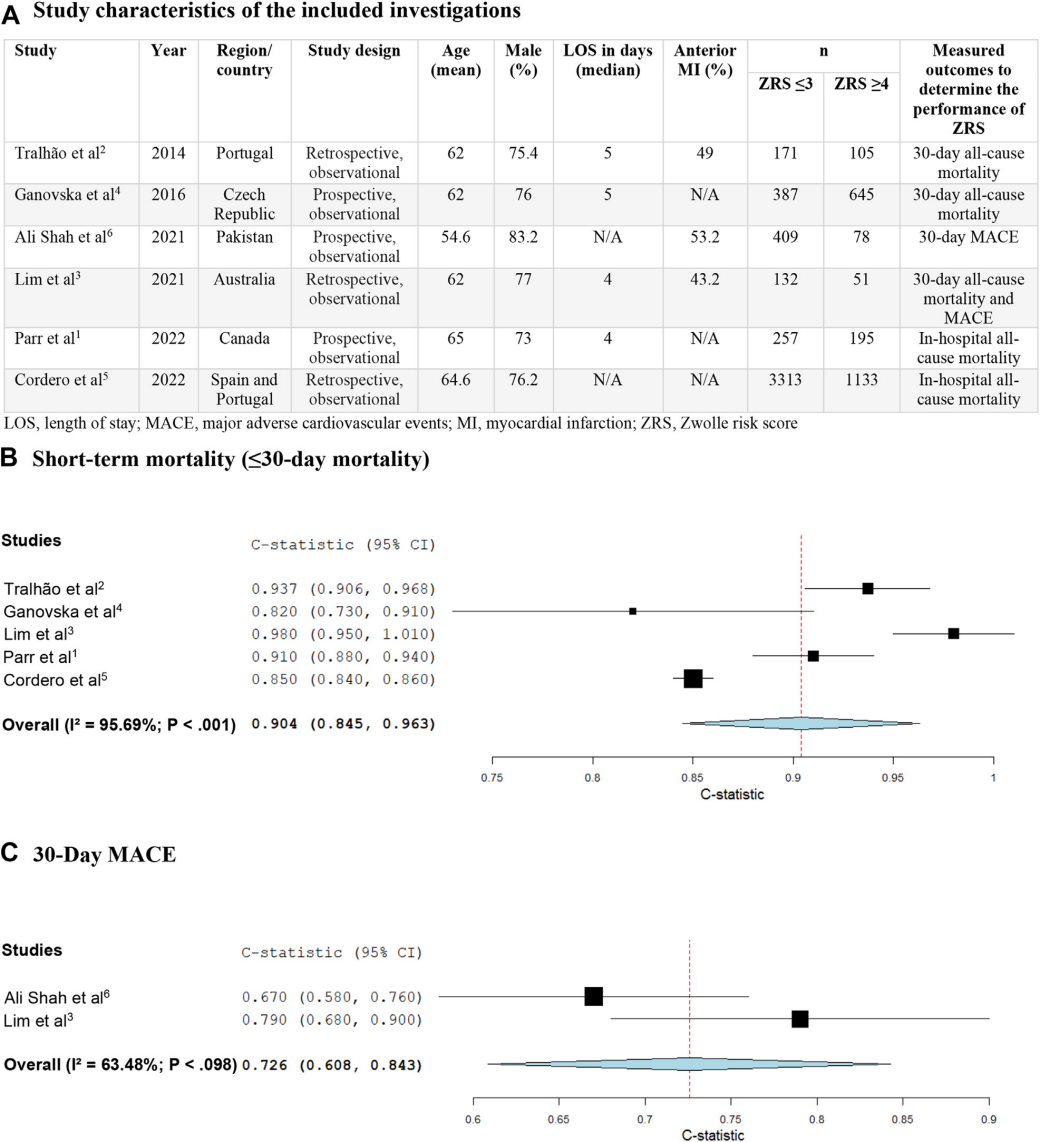
**(A) Study characteristics,(B) C-statistic for short-term mortality (pooled analysis),and (C) C-statistic for 30-day major acute cardiovascular events (MACE) (pooled analysis),in patients with STEMI**. LOS, length of stay; MI, myocardial infarction; STEMI, ST-segment elevation myocardial infarction; ZRS, Zwolle risk score.

Six observational studies were included, with 5 studies reporting short-term mortality^1^, ^2^, ^3^, ^4^, ^5^ and 2 studies reporting 30-day MACE.^3^^,^^6^ These studies included 6876 patients, with 4669 patients in the low-risk group (ZRS ≤3) and 2207 patients in the high-risk group (ZRS ≥4) (Figure 1A).^1^, ^2^, ^3^, ^4^, ^5^, ^6^ Included patients were mostly male (77%). Compared with the low-risk group, high-risk patients were older (67 vs 58 years) and had a higher prevalence of hypertension (62% vs 50%), diabetes (33% vs 25%), and a lower proportion of smokers (29% vs 47%). The ZRS showed excellent discriminating ability in predicting short-term mortality (n = 6389) (C-statistic, 0.9; 95% CI, 0.85-0.96; I^2^ = 96%; Figure 1B)^1^, ^2^, ^3^, ^4^, ^5^ and moderate discriminating ability in predicting 30-day MACE (n = 670) (C-statistic, 0.73; 95% CI, 0.61-0.84; I^2^ = 63%; Figure 1C)^3^^,^^6^ in STEMI patients.

In this meta-analysis of 6 studies, we found that, among patients with STEMI, the ZRS demonstrated a good predictive ability for short-term mortality and moderate predictive ability for the risk of 30-day MACE. To the best of our knowledge, this is the first meta-analysis on the applicability of the ZRS in the risk stratification of STEMI. In patients with STEMI, emergent PCI significantly decreases the ischemic burden and reduces the risk of ventricular arrhythmias and acute heart failure. Consequently, compared to the pre-PCI era, rates of in-hospital and postdischarge mortality have decreased in this population.^1^^,^^2^ In contemporary practice, several risk models are available to risk-stratify STEMI patients that can be used to guide early discharge or intensive care vs nonintensive care unit admissions.^1^^,^^3^ The ZRS is a quick, easy-to-use, well-validated tool that takes baseline patient characteristics and procedural aspects into account and adequately predicts adverse clinical outcomes.^1^, ^2^, ^3^ Although originally conceived to predict short-term mortality, but not MACE, the ZRS may serve health care systems as a triaging tool by optimizing the allocation of hospital resources and cardiac intensive care unit usage.^1^, ^2^, ^3^ Finally, the ZRS is not an all-encompassing scoring system, and aspects such as frailty, mobility, patient preferences, and multidisciplinary team input should be evaluated to optimize STEMI care.

This meta-analysis is limited by the small number of observational studies, the lack of patient-level data, variable definitions of MACE, and intrinsic limitations of the individual studies (including short follow-up and low event rates). In aggregate, the ZRS is a simple scoring method that showed strong predictive capacity for short-term mortality, but only moderate predictive ability for 30-day MACE in patients with STEMI.
